# Technical Considerations of Phosphorous-32 Bremsstrahlung SPECT Imaging after Radioembolization of Hepatic Tumors: A Clinical Assessment with a Review of Imaging Parameters

**DOI:** 10.1155/2014/407158

**Published:** 2014-03-27

**Authors:** Elahe Pirayesh, Mahasti Amoui, Shahram Akhlaghpoor, Shahnaz Tolooee, Maryam Khorrami, Hossain PoorBeigi, Shahab Sheibani, Majid Assadi

**Affiliations:** ^1^Department of Nuclear Medicine, Shohada-e-Tajrish Medical Center, Shahid Beheshti University of Medical Sciences, Tehran, Iran; ^2^Department of Interventional Radiology, Noor Medical Imaging Center, Tehran, Iran; ^3^Department of Nuclear Sciences, Iranian Atomic Energy Organization, Tehran, Iran; ^4^The Persian Gulf Nuclear Medicine Research Center, Bushehr University of Medical Sciences, Bushehr, Iran

## Abstract

*Background*. Bremsstrahlung (BS) imaging during radioembolization (RE) confirms the deposition of radiotracer in hepatic/extrahepatic tumors. The aim of this study is to demonstrate ^32^P images and to optimize the imaging parameters. * Materials and Methods*. Thirty-nine patients with variable types of hepatic tumors, treated with the intra-arterial injection of ^32^P, were included. All patients underwent BS SPECT imaging 24–72 h after tracer administration, using low energy high resolution (LEHR) (18 patients) or medium energy general purpose (MEGP) (21 patients) collimators. A grading scale from 1 to 4 was used to express the compatibility of the ^32^P images with those obtained from CT/MRI. * Results*. Although the image quality obtained with the MEGP collimator was visually and quantitatively better than with the LEHR (76% concordance score versus 71%, resp.), there was no statistically significant difference between them. * Conclusion*. The MEGP collimator is the first choice for BS SPECT imaging. However, if the collimator change is time consuming (as in a busy center) or an MEGP collimator is not available, the LEHR collimator could be practical with acceptable images, especially in a SPECT study. In addition, BS imaging is a useful method to confirm the proper distribution of radiotherapeutic agents and has good correlation with anatomical findings.

## 1. Introduction

Radioembolization (RE) is a promising therapeutic modality for patients with unresectable hepatic tumors. In this procedure, radioisotopes preferentially localize in the peritumoral and intratumoral arterial vasculature, while exposure to the normal hepatic parenchyma remains within tolerable limits. RE is based on the predominant arterial blood supply of hepatic tumoral lesions by the hepatic artery, in contrast to normal liver parenchyma, which is mainly supplied by the portal vein [1, 2]. Therapeutic agents are properly sized pharmaceuticals which incorporate the *β* radiating isotopes, such as ^90^Y, ^188^Re, and ^166^Ho [3–5]. The clinical applications of these *β*+ emitters for the treatment of different kinds of malignant and nonmalignant diseases are increasing [6]. Posttreatment imaging confirms the distribution of radiotracers within the target organ, or an additional unexpected deposition [7]. These data help the physician to predict the patient's response to RE therapy or the probable side effects [7].

In this study, phosphorus-32 (^32^P) particles were used for the RE of hepatic tumors. Phospherous-32, a pure *β*+ emitter available as a therapeutic radiopharmaceutical since the 1960s, has many suitable features for radioembolization therapy, such as a long half-life (14.3 days) and a maximum energy of 1.7 MeV. However, secondary photon emissions, called Bremsstrahlung (BS), produce a broad spectrum of limited energies and, therefore, compromise the selection of energy windows and collimation, as well as the reconstruction of the SPECT images [8, 9].

Most studies which assess the parameters for the BS imaging of pure *β* emitters (e.g., ^32^P or ^90^Y) have used phantoms [9–16], and there is a gap in the comprehensive research to evaluate these factors in clinical practice. This study was designed to optimize the imaging parameters, including the energy window and collimator type for BS imaging. In addition, we evaluated the correlation of this technique with the anatomical findings in patients with hepatic tumors being treated with RE.

## 2. Materials and Methods

A total of 39 patients with unresectable hepatic or metastatic tumors of any origin, which were candidates for RE [17] with ^32^P particles, were included in this study. The chromic phosphate ^32^P radiopharmaceutical, with a mean size of 50–150 *μ*m and a mean injected activity of 260 MBq (75–450 MBq), was produced locally at the research reactor (IAEO, Iran). Calculations of the appropriate radiation dose were undertaken according to the following formula: dose (Gy) = 7.3 × activity (mCi)/hepatic mass (kg) [18].

This study complies with the declaration of Helsinki and was approved by the institutional ethics committee of the Shahid Beheshti University of Medical Sciences, and all patients provided written informed consent.

### 2.1. Anatomical Imaging

Cross-sectional imaging with a CT or MRI was accomplished for all patients in order to determine the size and location of the lesions as numerical segments consistent with the physiological division of Couinaud [19]. The findings were reported by a radiologist, and the liver volume was calculated.

### 2.2. Radioembolization Procedure

The standard angiographic method for the assessment of the femoral artery was used [20]. The celiac and superior mesenteric arteries were catheterized using a Cordis Simon I catheter, and DSA angiography was prepared using a Siemens' C-Arm Angiography Unit. Superselective catheterization was done using Cook's 3F microcatheter and ^32^P particles were injected into the artery. Afterwards, a postembolization angiogram was obtained [21].

### 2.3. Bremsstrahlung Imaging

Imaging was conducted 24–72 hours after the RE of the hepatic tumors. The imaging system consisted of a Siemens single head e.cam gamma camera equipped with a low energy high resolution (LEHR) or a medium energy general purpose (MEGP) collimator. In order to select the window setting, the BS energy spectra with the LEHR and MEGP collimators were obtained using a 37 MBq ^32^P point source, in a glass vial placed at a distance of 10 cm from the collimator in the center of the field of view (FOV). An energy window setting of 100 keV ± 25% was chosen.

All patients were randomly divided into two groups, A and B. In group A, planar and SPECT imaging were done with the LEHR collimator, whereas an MEGP collimator was used in group B. Planar imaging was performed in the anterior and posterior projections of the upper abdomen for 10 minutes, using a 64 × 64 matrix. In the SPECT study, the data were acquired in a 64 × 64 matrix for 64 projections over 360°, for a period of 30 seconds per projection. Raw data were reconstructed from either filtered-back projections or iterative (ordered subsets expectation maximization (OSEM)) methods [22]. The OSEM method with four iterations and two subsets was selected as the method of choice for the reconstruction due to fewer image distortions. The images were reconstructed in transaxial, sagittal, and coronal slices.

### 2.4. Image Interpretation

The images obtained from the SPECT and CT/MRI were evaluated visually by two nuclear medicine specialists and one radiologist in a blinded and independent fashion. A linear black and white scale with a lower and upper threshold of 0% and 100%, respectively, was used for all planar and SPECT images. The distribution of ^32^P in the liver was assessed and reported as focal or multifocal lesions in the involved segments. Based on the compatibility of the ^32^P images with anatomical findings (CT/MRI), a grading system proposed by Boan et al. [23] was applied per patient as grade 4 for poor correlation, grade 3 for intermediate correlation, grade 2 for good correlation, and grade 1 for a perfect match. In cases of disagreement between the physicians, a consensus was reached. The extrahepatic activity was also reported.

### 2.5. Statistical Analysis

Continuous variables were expressed as the mean ± SD, and categorical variables were expressed as absolute values and percentages. A 2-tailed *t*-test was used to compare the mean values between the groups. The Mann-Whitney *U* test was used to compare the statistical differences between the LEHR and the MEGP collimators as a grading system. A *P* value of less than 0.05 was considered statistically significant. All statistical analyses were performed using an IBM computer and the SPSS Inc. PASW software, version 18.0.

## 3. Results

**Figure 1:**
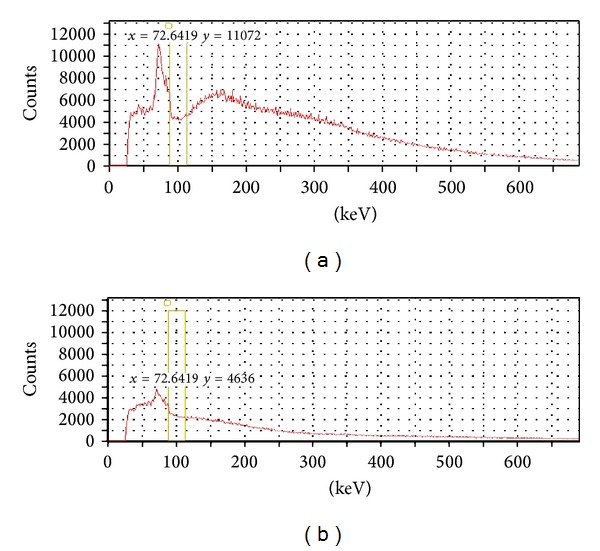
P-32 Bremsstrahlung energy spectra with (a) LEHR collimator and (b) MEGP collimator, obtained using a 37 MBq ^32^P point source, in a glass vial, at a distance of 10 cm from the collimator.

Thirty-nine patients with a variety of hepatic tumoral lesions were treated with RE and evaluated (Table 1). The choice of energy window shows the ^32^P BS energy spectra taken with a gamma camera equipped with the LEHR and MEGP collimators (Figure 1). In contrast to the standard gamma emitters, the energy spectrum of the BS is very complex, with no pronounced photopeak. The lowest detectable energy in the spectra is about 25 keV, with a peak of around 75 and 150 keV. Considering these spectra, the energy window was set at 100 keV ± 25% for all measurements. The number of measured photons was significantly reduced with the MEGP resulting in a reduced sensitivity.

**Figure 2:**
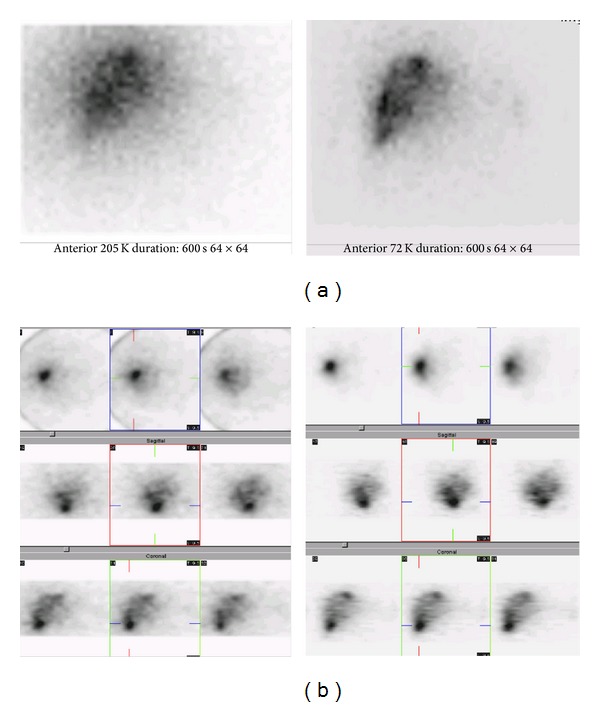
A patient with metastatic colon cancer treated with 333 MBq ^32^P particles. (a) Bremsstrahlung ³²P images in planar study with LEHR and MEGP collimators. (b) Bremsstrahlung ³²P images in SPECT study with LEHR and MEGP collimators.

**Figure 3:**
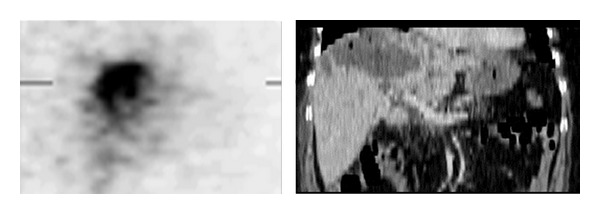
A patient with metastatic colon cancer treated with 296 MBq ^32^P particles. It shows a perfect concordance of Bremsstrahlung and CT images (grade 1).

**Figure 4:**
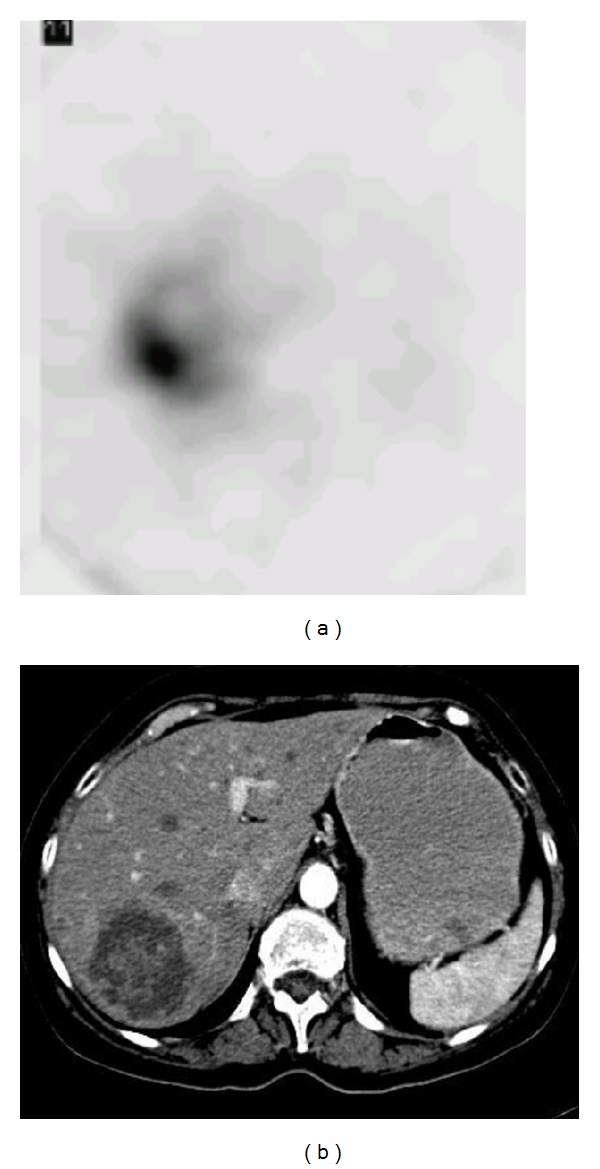
(a) Enlarged necrotic tissue secondary to previous chemoembolization (nonradioactive lipiodol) and (b) correlated CT slice.

Regarding the effect of the collimator type, 18 patients in group A were imaged with the LEHR collimator and 21 patients in group B, with the MEGP collimator. In addition, the planar and SPECT images from the LEHR and MEGP collimators were obtained (Figure 2). A comparison of the CT/MRI and BS SPECT images as the correlative grading score between the MEGP and LEAP collimators is summarized in Table 2. There is a good or perfect correlation with the anatomical findings (scores 1 and 2) in 13 (71%) BS images from group A (LEHR collimator), and in 16 (76%) from group B (MEGP collimator). However, this difference is not statistically significant (*P* value = 0.9). Taken as a whole, in 29 patients (74%) the BS images have a satisfactory concordance (Figure 3). A perfect concordance of the SPECT and CT images of a necrotic tumor is demonstrated in Figure 4.

**Figure 5:**
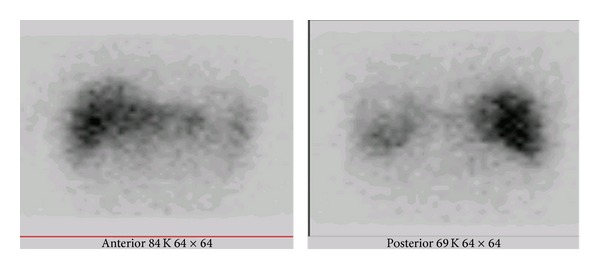
Unusual findings of splenic activity visualized after RE with ^32^P particles.

Some unusual findings were also recorded. The extrahepatic accumulation of the radiotracer was observed in the spleen of two patients, duodenum of one patient, and the pancreas of one patient (see Figure 5).

## 4. Discussion

The application of the *β*+ emitting radionuclides for the treatment of malignant and nonmalignant conditions is increasing [6]. In these cases, imaging can be performed by measuring the BS photons emitted from the *β* particles as they lose their energy in the body [24]. BS radiation is not ideal for diagnostic purposes, because of the continuous energy range, interseptal penetration of high energy photons, and the creation of photons far from the radiation emission site [9, 10]. Despite these problems, BS imaging is important in confirming the satisfactory delivery, pharmacokinetics, and potential abnormal deposition of the radiotracer [16, 25–29]. Furthermore, it has been demonstrated that BS imaging is useful for the direct quantification and dosimetry of *β*-emitter isotopes in clinical practice [4, 30–32]. We evaluated optimized parameters for ^32^P BS imaging after the RE of the hepatic tumors, and to our knowledge, this is the first clinical study to assess these factors to improve image quality.

As a result of the complexity of the spectrum and the absence of a pronounced photopeak, the task of selecting a suitable energy window was particularly difficult. Furthermore, the optimal energy window for a particular beta emitter is still a matter of debate within the research community [24]. Considering the ^32^P BS spectrum (Figure 1) and the results of other studies [26, 30, 33, 34], the energy window of 100 keV + 25% was selected. As shown in Figure 1, the lower energy (about 75 keV) of the ^32^P BS energy spectra is compatible with the X-ray characteristics due to the interaction of the high energy BS photons which lead the septa of the collimator. The rise in the spectrum of about 150 keV reflects the penetration of the septa of the collimators by higher energy. The same phenomenon has been previously reported in ^32^P, ^90^Y, and ^89^Sr BS measurements with a gamma camera [9–11, 13, 15, 27]. Although the maximum energies of ^32^P and ^90^Y are different (1.7 MeV vs. 2.27 MeV), their BS spectra appear to be similar. Ito et al. [11] compared different energy windows for BS ^90^Y imaging and concluded that images obtained with an energy window of 120 keV + 15% provided the highest resolution and lowest uncertainty. Shen et al. [9] showed that the best and most practical selection is an energy window of 55–285 keV. Therefore, the selection of the energy window varies with each study. Some researchers select narrow energy windows [16, 26, 34, 35], while others prefer wider ranges [7, 10, 29] depending on the purpose of the imaging; if the goal is accurate localization of distributed activity, a narrower range is ideal.

In this study, we examined the collimator type for ^32^P BS imaging. Thirty-nine patients were divided into two groups, with an LEHR collimator in group A and an MEGP collimator in group B. The other imaging parameters were similar in both groups. In comparison to the anatomical images, the concordance scores of the SPECT images in groups A and B were statistically insignificant. The MEGP collimator created higher quality images because of lower septal penetration background activity, compared to the LEHR. Meanwhile, the LEHR collimator did create acceptable images, particularly in the SPECT study (Figures 2 and 3). In a quantitative study on the planar images using the phantom, Shen et al. [9] showed that the sensitivity of the LEAP collimator was three times better than that of the MEGP collimator, whereas the system resolution of the MEGP collimator was three times better than that of the LEAP collimator. Similar results were obtained by Cipriani et al. [15] and Shukla et al. [13]. Therefore, most researchers working on BS imaging have used ME [7, 10, 11, 15, 25–30, 33–37] or HE collimators [16, 32].

In this study, we selected the narrow energy window and used the LEHR collimator (instead of the LEAP), which relatively improved the system resolution. In addition, compared to planar images, the SPECT method improves lesion contrast and anatomical clarity by the removal of superimposed radioactivity [38]. These factors influenced lesion detectability; thus, it could be concluded that the MEGP collimator is the best choice for BS SPECT imaging. However, if the collimator change is time-consuming (as in a busy center) or the MEGP collimator is not available, the LEHR collimator could be practical as it creates acceptable images, especially in SPECT studies.

Despite the inherently poor resolution of BS imaging, as our previous study showed, there is a relatively good correlation between BS imaging and the CT/MRI or other nuclear medicine studies [39]. Mansberg et al. [28] showed that BS images have anatomical correlations with sites containing maximum tumor density. Similarly, Tehranipour et al. [37] described concordant findings from the ^18^F-FDG PET and ^90^Y-Bremsstrahlung scans after the RE of a hepatic tumor in one case report. The correspondence of the ^99^mTc-MDP and ^89^Sr BS images was also reported [15, 35, 40].

Our study showed a relatively good correlation between the SPECT and CT/MRI images (Table 2). The results of this study reveal that BS SPECT images have adequate resolution for posttreatment evaluations, and that 74 percent of patients have good correlation or a perfect match with the CT/MRI images, confirming the technically appropriate localization of the radiopharmaceuticals. As such, a potential good response to therapy can be predicted. Conversely, a poor correlation could be due to small metastatic lesions undetectable in BS images because of an inherently low spatial resolution. Furthermore, the distribution of particles could be affected during intra-arterial injections by vessel selection, the flow in a selected vessel, or the size and amount of the injected particles. Discordant findings can also be related to technical errors (such as a superselective intra-arterial injection) during the RE, or poor vascularized hepatic tumors, thereby leading to the accumulation of radiotracers in normal tissues [26, 33, 41]. In contrast to concordance cases, the therapeutic response will not be ideal in discordant cases because of inadequate radiation to the target [26]. We will, however, evaluate the clinical applications of the anticipated grading system in an ongoing study.

The potential advantage of ^32^P scintigraphy is its ability to depict the vascularity of viable tumor cells rather than the necrotic tissue of a tumor mass (Figure 5). Therefore, BS imaging of P-32 after the RE of the hepatic tumors can be reflective of vascularized and viable tumoral tissues [33].

The extrahepatic deposition of radiotracers is an important finding, which can assist physicians in subsequent treatment planning. The extrahepatic activity in the spleen (Figure 4), lung, and GI tract can have probable unwanted complications. In addition, because of the lower delivered radiation dose to the target, failure in the treatment can be predicted. Ahmadzadehfar et al. demonstrated the importance of BS imaging to predict RE-induced extrahepatic side effects [29]. Sebastian et al. [26] have also reported gastric ulcerations as a result of microspheres entering into an aberrant gastric artery.

It should be mentioned that the current study had some limitations. One of the most important drawbacks is its small sample size. Another limitation is that we did not perform an angiogram with Tc-MAA to rule out a possible high lung shunt; however, further evidence needs to be acquired.

## 5. Conclusion

Despite the shortcomings of BS imaging, good quality images can be obtained by the optimization of the energy window and collimator type. BS imaging of ^32^P, after the RE of hepatic tumors, can confirm the hepatic and extrahepatic distribution of radiotracers to predict the patient's response to RE therapy. This study shows that an LEHR collimator may produce acceptable images, especially for the SPECT.

## Figures and Tables

**Table 1:** Characteristics of patients treated with RE.

Age	54 yr. (27–78)
Sex	*n* = 39
Male	20
Female	19
Primary cancer	
Colon	25
Breast	5
HCC	3
Carcinoid	3
Pancreas	1
Lung	2

**Table 2:** Correlation grading for compatibility of ^32^P images obtained by the LEHR collimator and the MEGP collimator, with anatomical findings.

	LEAP	MEGP	Total
Score 1	1	2	3
Score 2	4	3	7
Score 3	6	8	14
Score 4	7	8	15
Total	**18**	**21**	**39**
